# Swept-source optical coherence tomography angiography alleviates shadowing artifacts caused by subretinal fluid

**DOI:** 10.1007/s10792-020-01376-7

**Published:** 2020-04-24

**Authors:** Michael Reich, Daniel Boehringer, Kai Rothaus, Bertan Cakir, Felicitas Bucher, Moritz Daniel, Stefan J. Lang, Wolf A. Lagrèze, Hansjuergen Agostini, Clemens Lange

**Affiliations:** 1 Eye Center, Faculty of Medicine, University of Freiburg, Killianstrasse 5D-79106, Freiburg, Germany; 2 Faculty of Medicine, University of Freiburg, Freiburg, Germany; 3 Augenzentrum am St. Franziskus Hospital, Muenster, Germany

**Keywords:** OCT angiography, Choriocapillaris, Artifacts, Central serous chorioretinopathy, Spectral domain optical coherence tomography angiography, Swept-source optical coherence tomography angiography

## Abstract

**Purpose:**

To characterize the choriocapillaris (CC) structure in relation to subretinal fluid (SRF) as a possible systematic error source using spectral domain (SD-OCTA) compared to swept-source optical coherence tomography angiography (SS-OCTA).

**Methods:**

This is a prospective case-control study of 23 eyes. Ten patients with acute central serous chorioretinopathy (CSC), three patients with partial macular-off retinal detachment (RD) and ten healthy, age-matched controls were included. Abnormal CC decorrelation signals were quantitatively compared in CSC and controls by means of custom image processing. To investigate the influence of SRF on CC OCTA signal, the extent of SRF was quantified with a macular heatmap and compared with the corresponding OCTA signal of the CC.

**Results:**

SS-OCTA yielded a more homogeneous OCTA signal from the CC than SD-OCTA, offering less signal dispersion and variability in healthy and diseased eyes. Both devices demonstrated CC signal voids in CSC and RD, respectively. In CCS, the voids were predominantly located in the area with SRF. Compared to SD-OCTA, SS-OCTA delivered a more homogenous OCTA signal and reduced signal voids in the CC underneath SRF in both RD and CSC (CSC, 7.6% ± 6.3% vs, 19.7% ± 9.6%, *p* < 0.01). Despite this significant attenuation of signal voids, SS-OCTA continued to reveal signal voids below SRF and more pixels with reduced OCTA signals in CSC patients compared to controls (7.6% ± 6.3%, 0.1% ± 0.1%, *p* < 0.0001).

**Conclusion:**

Understanding OCTA artifacts is critical to ensure accurate clinical evaluations. In this study, we describe the presence of SRF as an important shadow-causing artifact source for CC OCTA analysis which can be mitigated but not completely eliminated by employing SS-OCTA.

## Introduction

The choriocapillaris (CC) is a few-µm-thin layer of capillaries of relatively large diameter located in the inner aspect of the choroid below the retinal pigmented epithelium (RPE) [1]. Changes in CC blood flow are known to occur physiologically with increasing age and are associated with a variety of chorioretinal diseases such as age-related macular degeneration (AMD) and central serous chorioretinopathy (CSC). While conventional imaging techniques such as fluorescein and indocyanine green angiography can only partially depict the CC, optical coherence tomography angiography (OCTA) enables us to assess and quantify the CC blood flow [2]. Briefly, OCTA technology employs motion contrast to image blood flow and thereby vessels through different segmented areas in the eye, thus eliminating the need for intravascular dyes. Since this technology’s introduction in 2014 [3], OCTA has developed rapidly and there are currently several generations of OCTA from different companies available. In the older-generation units, light is emitted by a spectral domain OCT (SD-OCT) with a wavelength of nearly 840 nm near the infrared range. The newer generations, on the other hand, apply swept-source OCT (SS-OCT) technology, which uses a longer wavelength of nearly 1050 nm (compared to 840 nm in SD-OCT) and a higher scan speed of 100,000 A-scans/s (compared to 68,000 A-scans/s). Thus, it allows deeper penetration into the tissue of 3 mm (compared to 2 mm), with the compromise of a slightly lower axial resolution of 6,3 µm (compared to 5 µm, personal communication with Zeiss).

Since OCTA is a light source-dependent technology, artifacts are very common and can lead to incorrect interpretations of OCTA images. They can arise from intrinsic characteristics of the eye, eye movements, OCTA image acquisition, image processing and display strategies [4]. In particular, shadow artifacts represent an important category of artifacts that can appear as a weakened signal behind an absorbing or scattering opacity or obstruction. Examples of shadow-causing artifacts are attenuated OCTA signals from the retinal vasculature behind media opacity [5] or retinal bleeding or a decreased OCTA signal from the CC caused by subretinal bleeding [6] or drusen [4]. Another potential artifact source is the presence of subretinal fluid (SRF), a common hallmark of various chorioretinal diseases [7]. SRF can appear in neovascular eye disease such as age-related or myopic macular degeneration, in RPE atrophy, such as in geographic atrophy in AMD, or in exudative disease of the CC such as choroidal tumor or pachychoroidal disease, including CSC [8].

While several studies have compared SD-OCTA and SS-OCTA in healthy patients [9, 10] and chorioretinal diseases [11], none has explicitly investigated the effects of SRF on the CC OCTA signal in both devices. Since SS-OCTA allows deeper light penetration and improved visualization of OCTA flow in deeper tissue [9], scattering and absorption caused by SRF might be reduced.

The aim of this study was therefore to compare the OCTA signal from the CC in health and disease and to investigate the effects of SRF on the CC OCTA signal with SD-OCTA and SS-OCTA. We demonstrate that SRF is an important shadow-causing artifact source in CC assessment that can be mitigated but not entirely eliminated when employing SS-OCTA.

## Patients and methods

### Study design

This was a prospective, observational, single-center, case-control study approved by our local ethics committee and adhering to the tenets of the Declaration of Helsinki.

### Study population

A total of 23 participants were included in this study. Ten patients (ten eyes) had acute CCS with SRF and three patients (three eyes) had retinal detachment (RD) with partial macular involvement. Ten age-matched healthy controls (10 eyes) with a visual acuity of at least 20/20 and no signs of chorioretinal pathology served as controls. All participants were examined in the Eye Center, University of Freiburg between June 2017 and August 2018. Patients presenting concomitant maculopathy, such as AMD, diabetic retinopathy, or other maculopathies were excluded. Patient characteristics such as gender, date of birth, preexisting conditions, initial diagnosis, prior treatment modalities and symptom duration were documented.

### Imaging and image analysis

OCTA images were taken with commercially available OCTA systems: the Zeiss Cirrus 5000 AngioPlex^®^ and Zeiss PLEX Elite 9000^®^. Zeiss Cirrus 5000 AngioPlex^®^ uses full spectrum spectral domain (SD-OCT) with a light source wavelength of 840 nm, an A-scan rate of 68,000 A-scans per second and an A-scan depth of 2.0 mm in tissue (1024 pixels). Zeiss PLEX Elite 9000® uses full spectrum swept source (SS-OCT) with a light source wavelength of 1050 nm, an A-scan rate of 100,000 A-scans per second and an A-scan depth of 3.0 mm in tissue (1536 pixels). Both OCTA-systems use the Optical Microangiography (OMAG^®^) algorithm to decorrelate signal detection. A real-time image-stabilizer (FastTracTM) ensured a minimum of movement artifacts. Furthermore, a built-in software was used to eliminate positive artifacts created from the superficial vascular layers. Each patient underwent a 6 × 6 mm^2^ volume scan of the CC layer. A 20 µm slab between 29 µm and 49 µm below the inner RPE was manually selected for each patient.

Abnormal CC decorrelation signals were quantified using a custom image processing algorithm programmed with “R” (www.r-project.org) as described previously [8, 12–14]. Briefly, images were processed via a Gaussian blur and morphological *h*-dome-operator, and the image’s average grayscale value was determined. The threshold for the color-coding was calculated for each OCTA image using the averaged grayscale value of all pixels (brightness of pixel) plus (increased flow, red pixels) or minus (decreased flow, green pixels) a constant and predefined threshold value. Before quantifying the pixel counts, shadowing artifacts of the inner retinal vessels were manually removed from each image. The pixel counts were used for statistical analyses of the CC signal’s homogeneity in SD-OCTA compared to SS-OCTA.

To analyze the influence of SRF on the OCTA CC signal, OCTA CC images were compared with the corresponding heatmap image provided by the OCTA devices. The area with a macular thickness exceeding 450 μm was defined as an area with significant SRF. Finally, the abnormal CC decorrelation signals were assessed in the area with or without SRF and statistically analyzed.

### Statistical methods

Statistical analysis was performed using GraphPad Prism 6 (GraphPad Software, Inc., La Jolla, CA, USA). The Mann–Whitney *U* test was used to compare two groups.

## Results

### Patient characteristics

A total of ten eyes from ten patients with acute CSC (male/female 6/4), three eyes from three patients with RD (male/female 2/1) and ten eyes from ten controls (male/female 4/6) were included. Average age was 56.1 years (range 38–77) in the CSC group, 58.7 years (range 51–63) in the RD group and 54.4 years (range 40–65) in the control group. Average visual acuity of the CSC patients ranged between 0.5 and 1.25, compared to a range of hand movement and 0.4 in the RD group and of 1.0 and 1.6 in the control group. Average time between initial diagnosis and examination of initial OCTA was 33.6 days (range 0–114) in the CSC group. After the initial diagnosis of acute CSC, four patients were treated with eplerenone, while six patients received no treatment.

### Homogeneity of choriocapillaris OCTA signal in SS-OCTA compared to SD-OCTA

**Fig.1:**
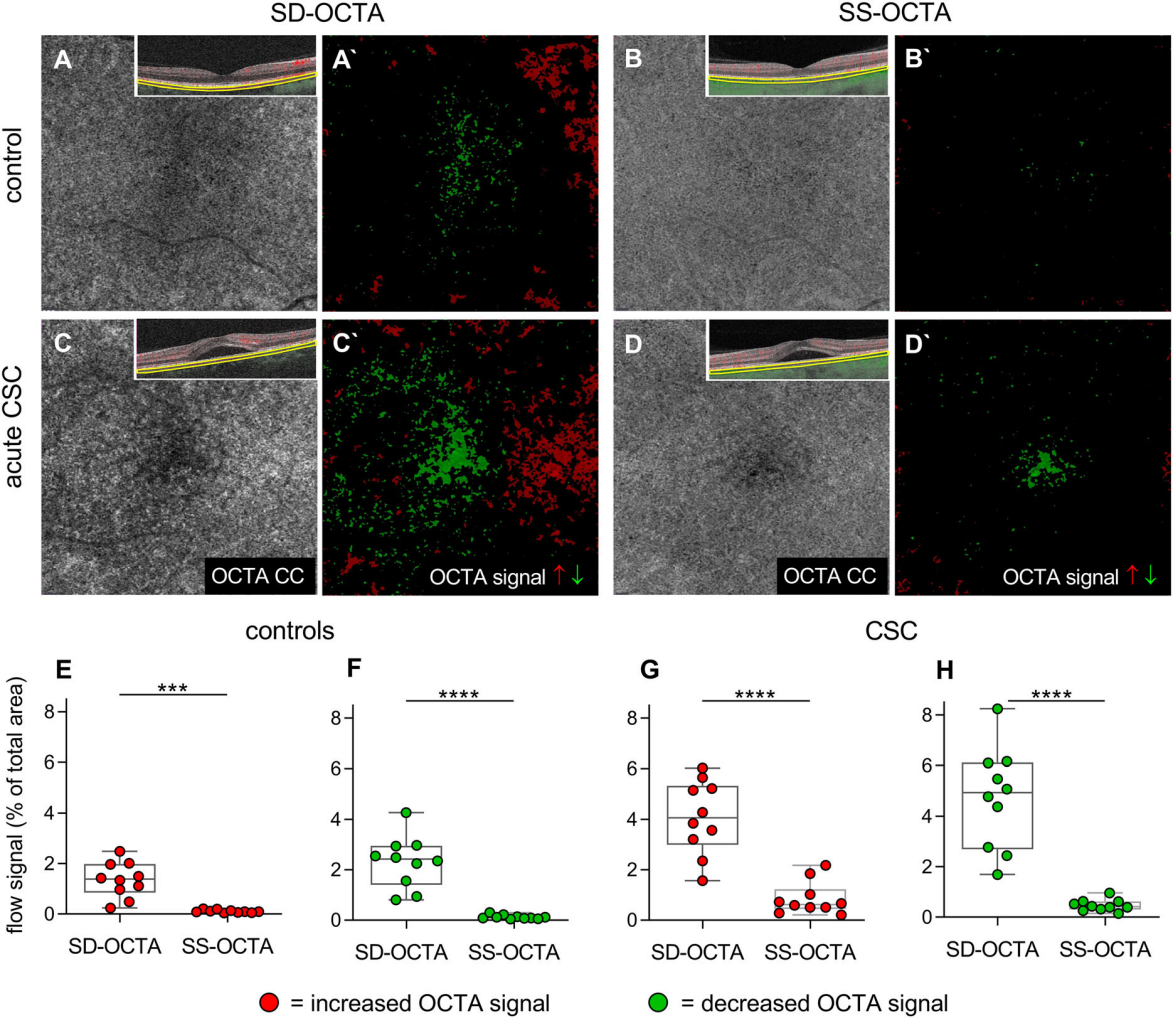
Comparison of spectral domain OCTA and swept- source OCTA in analyzing choriocapillaris architecture. A–D) Comparison of spectral domain OCTA (SD-OCTA) and swept-source OCTA (SS-OCTA) of the choriocapillaris (CC) in healthy controls (A, B, *n* = 10) and patients with acute central serous chorioretinopathy (CSC, C, D, *n* = 10). A′–D′) Increased CC OCTA signals are depicted in red; decreased OCTA signals are shown in green. E–H) Box and whiskers blots demonstrating the percentage of the total area of increased and decreased OCTA signal in healthy controls (E, F) and patients with acute CSC (G, H). Each dot represents one eye. Mann–Whitney *U*-test, ***p < 0.001 ****p < 0.0001

To examine the two OCTA devices with respect to their CC imaging, we first compared SS- to SD-OCTA CC images from healthy controls. SS-OCTA exhibited more a homogeneous CC architecture in all participants compared to SD-OCTA (Fig. 1A + B). When quantifying the number of pixels with increased or decreased OCTA signals from the choroid (Fig. 1A′ + B′), we found that SS-OCTA images revealed significantly fewer pixels with increased and decreased intensity than SD-OCTA images (*p* < 0.001 and *p* < 0.0001, respectively, Fig. 1E + F, Table 1).

**Table 1:** Homogeneity of choriocapillaris OCTA signal in swept- source OCTA compared to spectral domain OCTA

	SD-OCTA % of area (mean ± SD)	SS-OCTA % of area (mean ± SD)	*p* value
Controls			
Decreased flow	4.1 ± 1.5	0.8 ± 0.6	< 0.0001
Increased flow	1.4 ± 0.7	0.1 ± 0.1	< 0.001
CSC			
Decreased flow	4.7 ± 2.0	0.4 ± 0.2	< 0.0001
Increased flow	2.3 ± 1.0	0.1 ± 0.1	< 0.0001

Mann–Whitney *U* test was applied to compare two groups

*CSC* central serous chorioretinopathy, *SD-OCTA* spectral domain OCTA, *SS-OCTA* swept-source OCTA, *SD* standard deviation

Similarly, SS-OCTA images from patients with acute CSC were more homogeneous and revealed a more uniform pattern than SD-OCTA images (Fig. 1C+ D). The total area of pixels with an increased or decreased OCTA signal was significantly smaller when applying SS-OCTA compared to SD-OCTA (both *p* < 0.0001, Fig. 1G + H, Table1). Taken together, these data show that SS-OCTA provides a more homogeneous OCTA signal than SD-OCTA and less signal dispersion in both health and disease.

### Influence of subretinal fluid on the OCTA signal from the CC

**Fig. 2:**
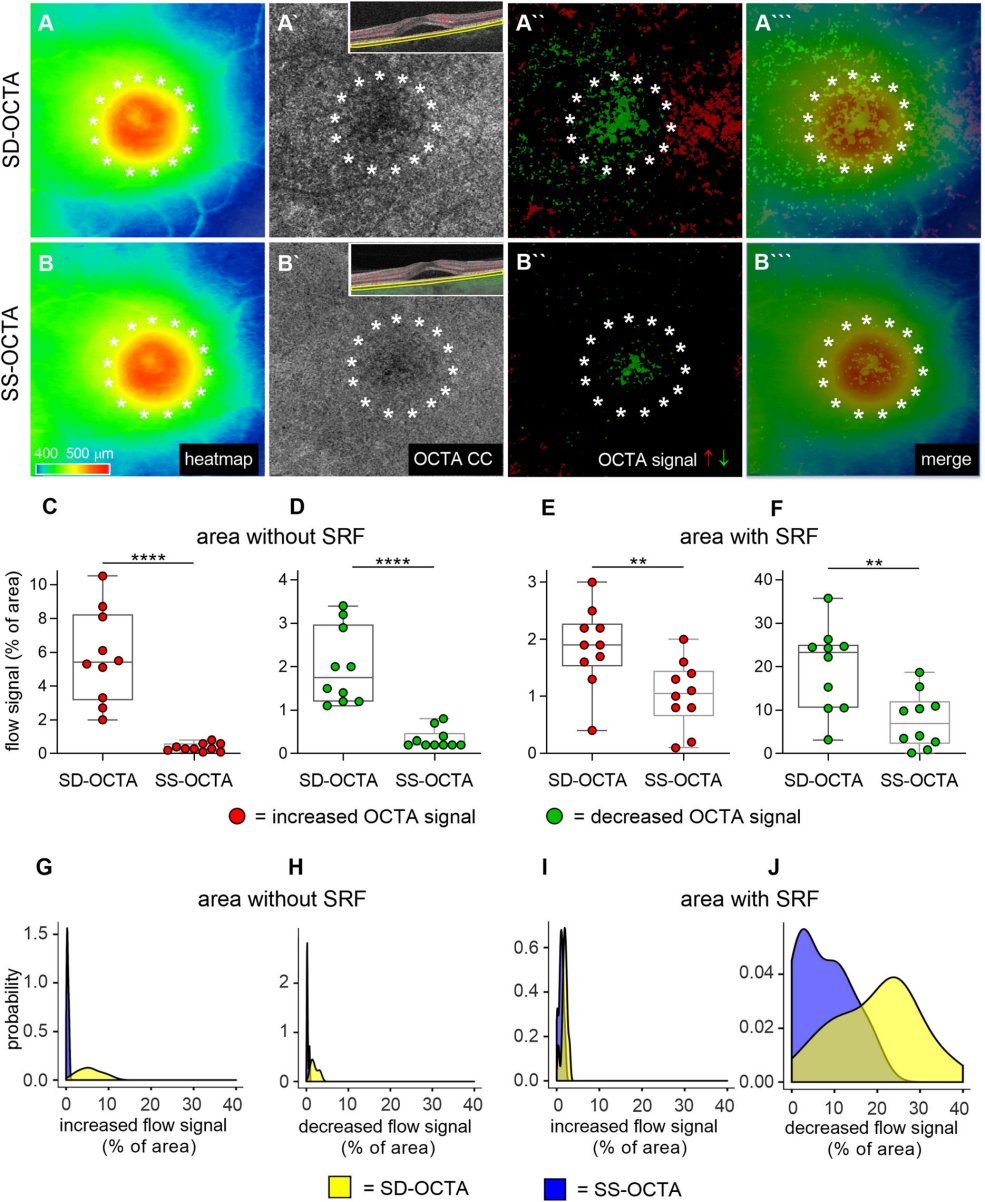
Influence of subretinal fluid on the OCTA flow signal from the choriocapillaris using spectral domain OCTA compared to swept- source OCTA. Comparison of spectral domain OCTA (SD-OCTA, A–A′″) and swept-source OCTA (SS-OCTA, B–B′″) signals at the level of the choriocapillaris (CC) in patients with acute central serous chorioretinopathy (CSC). Macular edema heatmap (A, B), corresponding OCTA image without (A′, B′) and with pseudocoloration (A″, B″) highlighting increased (red) and decreased OCTA signals (green) and a merged image (A′″, B′″) of a representative patient with acute CSC imaged with SD-OCTA (upper row) and SS-OCTA (bottom row). Asterisks surround the area of increased subretinal fluid (SRF) defined as > 450 µm macular thickness. C–F) Box and whisker plot demonstrating the relative number of increased (red) and decreased OCTA signal (green) in the area with (C, D) and without SRF (E, F) in SD-OCTA and SS-OCTA. Each dot represents one eye. Mann–Whitney *U*-test, **p < 0.01 ****p < 0.0001. G–J) Density plots of the OCTA choriocapillaris flow signal changes in the presence of SRF using SD-OCTA (yellow) compared to SS-OCTA (blue). G, H) Increased/decreased signal in the area without SRF, I, J) Increased/decreased signal in the area with SRF. The *x*-axis describes the density of flow signal changes in the respective area, the *y*-axis the probability of the corresponding percentage. Note that the distributions of SD-OCTA are uniformly broader in comparison with the distribution from SS-OCTA: This hints towards a better signal-to-noise ratio from the latter imaging modality

To compare SD-OCTA and SS-OCTA CC-images with respect to the presence or absence of SRF, we analyzed SD- and SS-OCTA images and their corresponding edema heatmaps from patients with acute CSC (Fig. 2, Table 2).

**Table 2:** Influence of subretinal fluid on the OCTA flow signal from the choriocapillaris using spectral domain OCTA compared to swept- source OCTA

	SD-OCTA % of area (mean ± SD)	SS-OCTA % of area (mean ± SD)	*p* value
CSC area with SRF			
Decreased flow	19.7 ± 9.6	7.6 ± 6.3	< 0.01
Increased flow	1.9 ± 0.7	1.0 ± 0.6	< 0.01
CSC area w/o SRF			
Decreased flow	2.0 ± 0.9	0.3 ± 0.2	< 0.0001
Increased flow	5.7 ± 2.7	0.4 ± 0.2	< 0.0001
Controls			
Decreased flow	2.3 ± 1.0	0.1 ± 0.1	< 0.0001
Increased flow	1.7 ± 0.7	0.1 ± 0.1	< 0.001

Mann–Whitney *U* test was applied to compare two groups

*CSC* central serous chorioretinopathy, *SRF* subretinal fluid, *SD-OCTA* spectral domain OCTA, *SS-OCTA* swept-source OCTA, *SD* standard deviation

In line with our results from our healthy cohort, we found that SS-OCTA yields a more homogeneous OCTA signal from the CC than does SD-OCTA in patients with CSC. When quantifying pixel numbers with increased or decreased CC OCTA signal outside the area of SRF, SS-OCTA images detected significantly fewer increased and decreased pixels compared to SD-OCTA (Fig. 2C + D, Table 2). Similarly, SS-OCTA resulted in a more homogeneous OCTA signal and reduced signal voids in the area with SRF compared to SD-OCTA. While the number of increased OCTA signal pixels was low in the area with SRF regardless of which device was used (Fig. 2E, Table 2), SS-OCTA resulted in a significant reduction in the number of pixels with a reduced OCTA signal compared to SD-OCTA (*p* < 0.01, Fig. 2F, Table 2). Despite this significant attenuation of signal voids in the CC, SS-OCTA still depicted signal voids underneath the SRF and more pixels with reduced OCTA signals in patients with CSC compared to healthy controls (*p* < 0.0001, Table 2).

Next, we depicted the distribution of OCTA flow signal changes in the area with or without SRF in both devices using histograms (Fig. 2G–J). In line with the above results, SD-OCTA generally showed more fluctuating OCTA changes, resulting in a broader histogram and thus indicating significant variability. SS-OCTA, on the other hand, resulted in less variability of OCTA CC signal changes compared to SD OCTA as represented by steeper histograms. However, the variability of pixels with decreased signal intensity (i.e., signal voids) in the area with SRF remained considerable compared to the area without SRF, indicating disease-associated changes in the CC of patients with CSC which may be reduced but not completely eliminated by employing SS-OCTA.

**Fig. 3:**
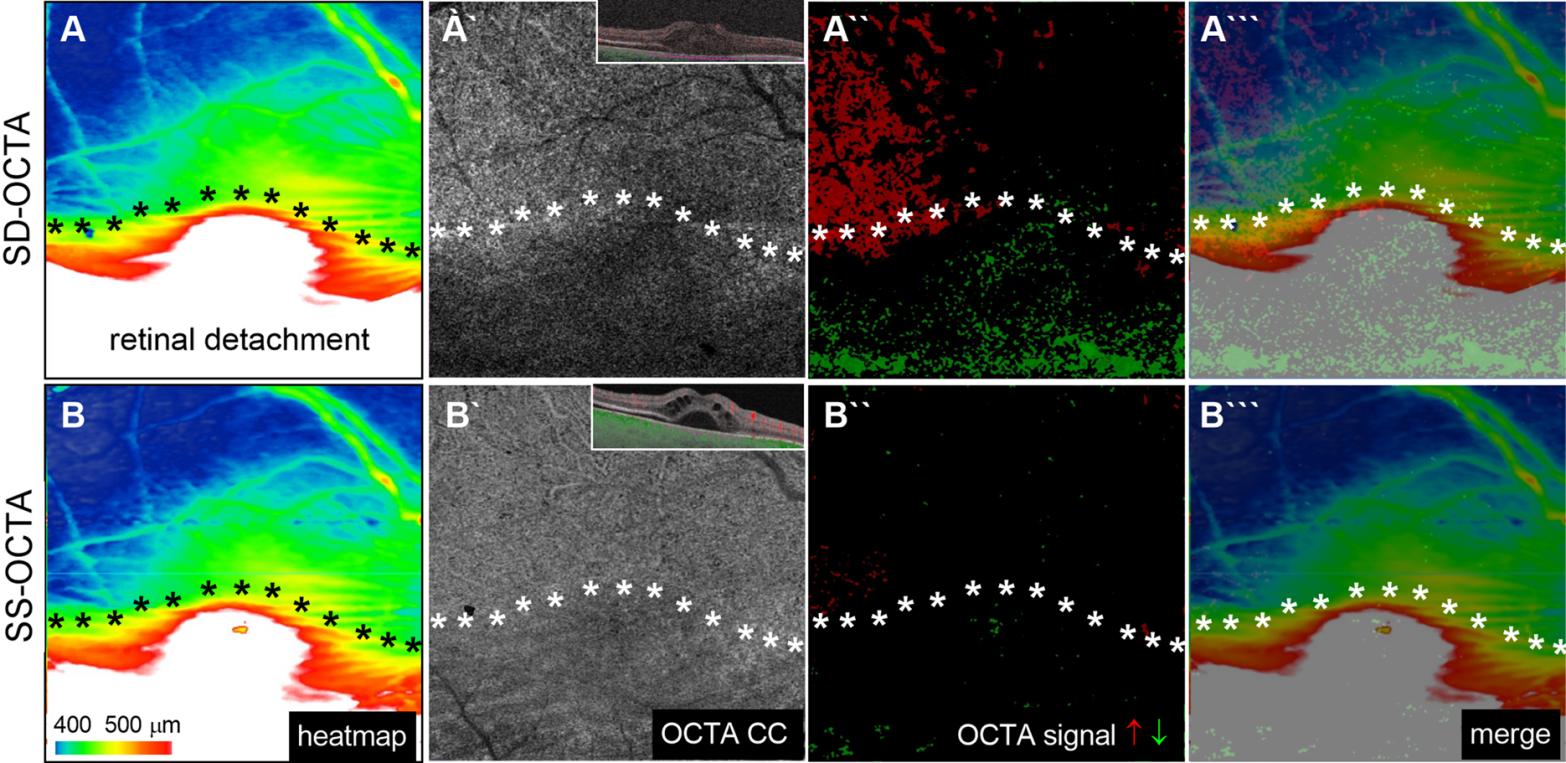
Comparison of the choriocapillaris flow signal in spectral domain OCTA (SD-OCTA) and swept- source OCTA (SS-OCTA) in a patient with macular off retinal detachment. Comparison of the spectral domain spatial distribution of choriocapillaris (CC) OCTA-signals detected with spectral domain OCTA (SD-OCTA, upper row) and swept-source OCTA (SS-OCTA, bottom row) in relation to the presence or absence of subretinal fluid in patients with partial macular off retinal detachment. Macular edema heatmap (A, B), corresponding OCTA image without (A`, B`) and with pseudocoloration (A``, B``) highlighting increased (red) and decreased OCTA signals (green) and a merged image (A```, B```). Asterisks outline the area of SRF defined as > 450 µm macular thickness

To generalize our previous observations on the influence of SRF in CC OCTA analysis and to explore disease-independent phenomena, we next investigated the CC architecture in patients with acute rhegmatogenous, partially macular-off retinal detachment (Fig. 3). In line with the aforementioned results, SD-OCTA revealed a reduced OCTA signal in the area with SRF which was less pronounced using SS-OCTA. However, the SS-OCTA signal from the CC in the SRF range remained reduced compared to the area without SRF in the same eye. This difference was less pronounced than in SD-OCTA and underlines our above-mentioned hypothesis that SS-OCTA technology reduces shadow artifacts by SRF but does not completely eliminate them.

## Discussion

Understanding OCTA artifacts is essential to ensure accurate clinical evaluation. In this study, we describe the presence of SRF as an important shadow-causing artifact source when assessing the CC—one that can be mitigated but not completely eliminated when employing swept-source OCTA.

We show that SRF is associated with a disease-independent, reduced SD-OCTA signal from the CC by examining patients with acute CSC and RD. Our results are consistent with previous SD-OCTA studies describing a reduced CC flow signal in patients with CSC and SRF [8, 12, 13, 15–17]. Similarly, numerous investigations have reported dark regions in OCTA CC scans, the so-called flow voids, in association with various chorioretinal diseases such as diabetic retinopathy [18], age-related macular degeneration [19] and CSC [8, 17] suggesting that they represent potentially disease-relevant hypoperfusion in the CC. However, the comparison of SS-OCTA and SD-OCTA in our study suggests that detecting a reduced CC OCTA signal in the presence of SRF with SD-OCTA is at least partially artificial and may have been over-interpreted in the past. SS-OCTA led to a more homogeneous OCTA CC architecture in health and disease and to a decline in the diminished OCTA signal in the SRF range compared to SD-OCTA. This is attributable to the use of a longer wavelength, which reduces scattering and absorption caused by SRF, thus allowing deeper light penetration and improved visualization of OCTA flow in deeper tissue [10, 20].

Several studies have been conducted comparing SD with SS-OCT imaging [5, 10, 20, 21], all showing an advantage of SS-OCT when imaging beyond the retinal layers. Compared to the other mentioned studies, our study distinguishes between areas with and without SRF. We could therefore demonstrate that the OCTA signal strength in areas with SRF is reduced compared to areas without SRF in SD- and SS-OCTA, suggesting that SRF is a critical artifact source in CC imaging. Subretinal fluid, with its often protein-rich content, can lead to increased absorption or scattering of OCTA light, leading thus to reduced signal recognition by CC. This finding is supported by evidence from other imaging technologies such as fundus autofluorescence revealing reduced autofluorescence corresponding to the areas of SRF accumulation in 82.3% of patients with CSC [22].

Interestingly, while SS-OCT can minimize the shadowing artifact caused by SRF, patients with acute CSC still exhibit a reduced OCTA flow signal in the CC. This can either be attributed to a diminished but persistent shadowing artifact or alternatively hypoperfusion of the CC in patients with CSC. The latter would be in line with studies demonstrating a reduced OCTA flow in patients with CSC [8, 17, 23, 24]—potentially the result of low blood flow [25, 26], defective CC endothelial cells [27, 28] or a thinned CC being pushed upward by pachyvessels accompanied by enlarged vascular space in Haller’s layer [17, 24, 29, 30]. Nicolò et al. [31] examined quantitative changes in choroidal flow areas using SS-OCTA and reported on dark patterns in the choroid regardless of the amount of fluid in CSC or in fellow eyes—anomalies not observed in healthy eyes. Similarly, we recently demonstrated reduced OCTA signals in previous SRF areas in patients with inactive CSC indicating reduced CC blood flow in patients with CSC [8]. Despite this evidence, it is currently not possible to state whether the reduced OCTA signal in patients with CSC is artificial or caused by disease-associated vascular changes. Further studies employing even better imaging modalities are warranted to elucidate this issue with confidence.

One of our study’s limitations is its small patient cohort. However, we consider it acceptable because of the robust signal strength when comparing SD- and SS-OCTA and the low interindividual standard deviation. Another limitation of our study is that we relied on the averaged pixel intensity of each image to determine increased and decreased flow changes within the same image, which may have caused some over- and underestimations of flow changes. Prospective longitudinal studies employing optimized image acquisition techniques less prone to artifact are therefore necessary to further validate our findings.

Taken together, our data provide evidence that the presence of SRF is an important shadowing artifact source for CC assessment that can be mitigated but not entirely eliminated when using SS-OCTA. SS-OCTA therefore appears to be superior to SD-OCTA for acquiring structural and flow information from deeper tissues such as the CC. Although SS-OCTA reduces the shadow artifacts caused by SRF, signal voids are still detected in CC from patients with CSC, which can be attributed to a reduced but persistent shadow artifact or hypoperfused CC. Further studies employing even better imaging technology and entailing OCTA follow-up of patients are needed to answer this question with certainty.

## Data Availability

More data if necessary are available from the corresponding author on reasonable request.
